# Supplementary material for: Microgreens: nutritional properties, health benefits, production techniques, and food safety risks
Source: PeerJ. 2025 Nov 25;13:e17938. doi: 10.7717/peerj.17938 (PMC12662059; doi:10.7717/peerj.17938)
Supplement: Supplemental Information 1 [file peerj-13-17938-s001.docx]

**References**

ALLOGGIA FP, BAFUMO RF, RAMIREZ DA, MAZA MA, CAMARGO AB. (2023). *Brassicaceae* microgreens: a novel and promissory source of sustainable bioactive compounds. *Curr Res Food Sci,* **10**(6):100480.

APPOLLONI E, PENNISI G, ZAULI I, CAROTTI L, PAUCEK I, QUAINI S, ORSINI F, GIANQUINTO G. (2022). Beyond vegetables: effects of indoor led light on specialized metabolite biosynthesis in medicinal and aromatic plants, edible flowers, and microgreens. *J Sci Food Agric,* **102**(2):472-487.

ARTÉS-HERNÁNDEZ F, CASTILLEJO N, MARTÍNEZ-ZAMORA L. (2022). Uv and visible spectrum led lighting as abiotic elicitors of bioactive compounds in sprouts, microgreens, and baby leaves-a comprehensive review including their mode of action. *Foods,* **11**(3):265.

ARYA KS, SANGEETA KUTTY M, PRADEEPKUMAR T. (2023). Microgreens of tropical edible-seed species, an economical source of phytonutrients- insights into nutrient content, growth environment and shelf life. *Future Foods*, **8**:1000262.

AYENI A. (2021). Nutrient content of micro/baby-green and field-grown mature foliage of tropical spinach (amaranthus sp.) and roselle (hibiscus sabdariffa l.). *Foods,* **10**(11):2546.

BERGŠPICA I, OZOLA A, MILTIŅA E, ALKSNE L, MEISTERE I, CIBROVSKA A, GRANTIŅA-IEVIŅA L. (2020). Occurrence of pathogenic and potentially pathogenic bacteria in microgreens, sprouts, and sprouted seeds on retail market in riga, latvia. *Foodborne Pathog Dis*, **17**(7):420-428.

BHASWANT M, SHANMUGAM DK, MIYAZAWA T, ABE C, MIYAZAWA T. (2023). Microgreens-a comprehensive review of bioactive molecules and health benefits. *Molecules,* **28**(2):867.

BHASWANT M, MIYAZAWA T, ABE C, FUKASAWA C, HIGUCHI O, THI MTN, MIYAZAWA T. (2024). Comparative analysis of macro- and micro-nutrients of Perilla frutescens var. crispa f. viridis microgreens and germinated seeds. *Food Chemistry,* **455**: 139858.

BRAZAITYTĖ A, VIRŠILĖ A, SAMUOLIENĖ G, VAŠTAKAITĖ-KAIRIENĖ V, JANKAUSKIENĖ J, MILIAUSKIENĖ J, NOVIČKOVAS A, DUCHOVSKIS P. (2019). Response of mustard microgreens to different wavelengths and durations of uv-a leds. *Front Plant Sci,* **10**:1153.

BUCKY A, PICMANOVA M, PORLEY V, PONT S, AUSTIN C, KHAN T, MCDOUGALL G, JOHNSTONE A, STEWART D. (2024). Light manipulation as a route to enhancement of antioxidant properties in red amaranth and red lettuce. *Front Nutr,* 11:1386988.

CASTELLANETA A, HORING M, LOSITO I, LEONI B, SANTAMARIA P, CALVANO CD, CATALDI TRI, MATYSIK S, LIEBISCH G. (2024). Exploration of the lipid profile of edible oleaginous microgreens by mass spectrometry-based lipidomics. *J Agric Food Chem,* **72**(20):11438-11451.

CHEN M, CHORY J. (2011). Phytochrome signaling mechanisms and the control of plant development. *Trends in Cell Biology,* **21**(11):664-671.

DE FRANCESCO S, LE DISQUET I, PEREDA-LOTH V, TISSEYRE L, DE PASCALE S, AMITRANO C, CARNERO DIAZ E, DE MICCO V. (2024). Combined effects of microgravity and chronic low-dose gamma radiation on brassica rapa microgreens. *Plants (Basel),* 14(1):64.

DURAIRAJAN BM, VELAVAN SUNDARARAJAN V, KANNAN G, MATHEWS PAUL B, MUNIYANDI K, THANGARAJ P. (2024). Elicitation of nutritional, antioxidant, and antidiabetic potential of barnyard millet (Echinochloa esculenta (A. Braun) H. Scholz) sprouts and microgreens through in vitro bio-accessibility assessment. *Food Chem*, **441**:138282.

EBERT AW. (2022). Sprouts and microgreens-novel food sources for healthy diets. *Plants (Basel),* **11**(4):571.

FAYEZIZADEH MR, ANSARI NA, SOURESTANI MM, FUJITA M, HASANUZZAMAN M. (2024). Management of secondary metabolite synthesis and biomass in basil (ocimum basilicum l.) microgreens using different continuous-spectrum led lights. *Plants (Basel),* 13(10):1394.

EL-NAKHEL C, PANNICO A, GRAZIANI G, KYRIACOU MC, GIORDANO M, RITIENI A, DE PASCALE S, ROUPHAEL Y. (2020). Variation in macronutrient content, phytochemical constitution and in vitro antioxidant capacity of green and red butterhead lettuce dictated by different developmental stages of harvest maturity. *Antioxidants (Basel),* **9**(4):300.

FRĄSZCZAK B, KLEIBER T. (2022). Microgreens biometric and fluorescence response to ıron (fe) biofortification. *Int J Mol Sci,* **23**(23):14553.

FUENTE B, LÓPEZ-GARCÍA G, MÁÑEZ V, ALEGRÍA A, BARBERÁ R, CILLA A. (2020). Antiproliferative effect of bioaccessible fractions of four *brassicaceae* microgreens on human colon cancer cells linked to their phytochemical composition. *Antioxidants (Basel),* **9**(5):368.

FUZAWA M, DUAN J, SHISLER JL, NGUYEN TH. (2021). Peracetic acid sanitation on arugula microgreens contaminated with surface-attached and ınternalized tulane virus and rotavirus. *Food Environ Virol,* **13**(3):401-411.

GARCIA-PEREZ P, TOMAS M, GIUBERTI G, CAPANOGLU E, CALLEGARI ML, LUCINI L, PATRONE V. (2025). Brassica microgreens shape gut microbiota and functional metabolite profiles in a species-related manner: a multi-omics approach following in vitro gastrointestinal digestion and large intestine fermentation. *Microbiol Res,* 298:128226.

GAO M, HE R, SHI R, ZHANG Y, SONG S, SU W, LIU H. (2021). Differential effects of low light intensity on broccoli microgreens growth and phytochemicals. Agronomy, **11**(3):537.

GHOORA MD, SRIVIDYA N. (2020). Effect of packaging and coating technique on postharvest quality and shelf life of raphanus sativus l. and hibiscus sabdariffa l. microgreens. *Foods,* **9**(5):653.

GUDZİNSKAITE I, LAUZIKE K, PUKALSKAS A, SAMUOLIENE G. (2024). The effect of light ıntensity during cultivation and postharvest storage on mustard and kale microgreen quality. *Antioxidants (Basel)*, 13(9):1075.

GUPTA A, SHARMA T, SINGH SP, BHARDWAJ A, SRIVASTAVA D, KUMAR R. (2023). Prospects of microgreens as budding living functional food: Breeding and biofortification through omics and other approaches for nutritional security. *Front Genet,* **14**:1053810.

HUANG H, JIANG X, XIAO Z, YU L, PHAM Q, SUN J, CHEN P, YOKOYAMA W, YU LL, LUO YS, WANG TT. (2016). Red cabbage microgreens lower circulating low-density lipoprotein (ldl), liver cholesterol, and inflammatory cytokines in mice fed a high-fat diet. *J Agric Food Chem,* **64**(48):9161-9171.

ISIK S, CETIN B, TOPALCENGIZ Z. (2024). Transfer of Salmonella, Escherichia coli O157:H7 and Listeria monocytogenes from contaminated soilless substrate and seeds to microgreens. *Int J Food Microbiol*, **414:**110612.

JAMBOR T, GOC Z, ZUSCIKOVA L, GREIFOVA H, KOVACIK A, KOVACIKOVA E, PEC M, LUKAC N. (2025). Phytochemical screening and monitoring of intercellular changes in murine leydig cells after the treatment of trigonella foenum-graecum l. microgreens in vitro. *Physiol Res,* 74(1):115-128.

JAUREGUI MJ, WARREN ER, DI GIOIA F, KWASNIEWSKI MT, LAMBERT JD. (2025). Effects of hot air drying on the nutritional and phytochemical composition of radish (raphanus sativus l.) microgreens. *J Food Sci,* 90(7):e70426.

KATHI S, LAZA H, SINGH S, THOMPSON L, LI W, SIMPSON C. (2023). Vitamin c biofortification of broccoli microgreens and resulting effects on nutrient composition. *Front Plant Sci,* **14**:1145992.

KHATTAB MS, ALY TAA, MOHAMED SM, NAGUIB AMM, AL-FARGA A, ABDEL-RAHIM EA. (2022). Hordeum vulgare l*.* microgreen mitigates reproductive dysfunction and oxidative stress in streptozotocin-induced diabetes and aflatoxicosis in male rats. *Food Sci Nutr,* **10**(10):3355-3367.

KIM SH, DHUNGANA SK, KIM ID, ADHIKARI A, KIM JH. Effect of illite treatment on quality characteristics and antioxidant activity of broccoli (brassica oleracea l. var. italica) sprouts. *Molecules,* 29(18):4347.

KYRIACOU MC, EBERT AW, SAMUOLIENĖ G, BRAZAITYTĖ A. (2022). Editorial: sprouts, microgreens and edible flowers: modulation of quality in functional specialty crops. *Front Plant Sci,* **13**:1033236.

KYRIACOU MC, EL-NAKHEL C, PANNICO A, GRAZIANI G, SOTERIOU GA, GIORDANO M, PALLADINO M, RITIENI A, DE PASCALE S, ROUPHAEL Y. (2020). Phenolic constitution, phytochemical and macronutrient content in three species of microgreens as modulated by natural fiber and synthetic substrates. *Antioxidants (Basel),* **9**(3):252.

LEE YM, YOON Y, YOON H, PARK HM, SONG S, YEUM KJ. (2017). Dietary anthocyanins against obesity and inflammation. *Nutrients,* **9**(10):1089.

LI X, TIAN S, WANG Y, LIU J, WANG J, LU Y. (2021). Broccoli microgreens juice reduces body weight by enhancing insulin sensitivity and modulating gut microbiota in high-fat diet-induced C57BL/6J obese mice. *Eur J Nutr,* **60**(7):3829-3839.

LIU K, GAO M, JIANG H, OU S, LI X, HE R, LI Y, LIU H. (2022). Light intensity and photoperiod affect growth and nutritional quality of brassica microgreens. *Molecules,* **27**(3):883.

LU Y, DONG W, YANG T, LUO Y, CHEN P. (2021). Preharvest uvb application increases glucosinolate contents and enhances postharvest quality of broccoli microgreens. *Molecules,* **26**(11):3247.

MA S, TIAN S, SUN J, PANG X, HU Q, LI X, LU Y. (2022). Broccoli microgreens have hypoglycemic effect by improving blood lipid and inflammatory factors while modulating gut microbiota in mice with type 2 diabetes. *J Food Biochem,* **46**(7):e14145.

MARCHIONI I, MARTINELLI M, ASCRIZZI R, GABBRIELLI C, FLAMINI G, PISTELLI L, PISTELLI L. (2021). Small functional foods: comparative phytochemical and nutritional analyses of five microgreens of the brassicaceae family. *Foods,* **10**(2):427.

MICHELL KA, ISWEIRI H, NEWMAN SE, BUNNING M, BELLOWS LL, DINGES MM, GRABOS LE, RAO S, FOSTER MT, HEUBERGER AL, PRENNI JE, THOMPSON HJ, UCHANSKI ME, WEIR TL, JOHNSON SA. (2020). Microgreens: consumer sensory perception and acceptance of an emerging functional food crop. *J Food Sci,* **85**(4):926-935.

MIR SA, SHAH MA, MIR MM. (2017). Microgreens: production, shelf life, and bioactive components. *Crit Rev Food Sci Nutr,* **57**(12):2730-2736.

MOHAMED SM, ABDEL-RAHIM EA, ALY TA, NAGUIB AM, KHATTAB MS. (2022). Barley microgreen incorporation in diet-controlled diabetes and counteracted aflatoxicosis in rats. *Exp Biol Med* (Maywood), **247**(5):385-394.

MORARU PI, RUSU T, MINTAS OS. (2022). Trial protocol for evaluating platforms for growing microgreens in hydroponic conditions. *Foods,* **11**(9):1327.

NAKAKAAWA L, GBALA ID, CHESETO X, BARGUL JL, WESONGA JM. (2023). Oral acute, sub-acute toxicity and phytochemical profile of brassica carinata a. braun microgreens ethanolic extract in wistar rats. *J Ethnopharmacol,* **305**:116121.

NAKAKAAWA L, GBALA ID, BARGUL JL, CHESETO X, WESONGA JM. (2025). Therapeutic potential of brassica carinata microgreens extract in alleviating symptoms of type 2 diabetes in wistar rats. *Food Sci Nutr,* 13(1):e4635.

NAUSHAD S, MATHEWS A, DUCEPPE MO, KANG M, WANG LR, HUANG H. (2022). Complete genome sequences of three listeria monocytogenes strains from microgreens obtained with minion and miseq sequencing. *Microbiol Resour Announc,* **11**(7):e0027722.

NEWMAN RG, MOON Y, SAMS CE, TOU JC, WATERLAND NL. (2021). Biofortification of sodium selenate ımproves dietary mineral contents and antioxidant capacity of culinary herb microgreens. *Front Plant Sci,* **12**:716437.

PARADISO VM, CASTELLINO M, RENNA M, GATTULLO CE, CALASSO M, TERZANO R, ALLEGRETTA I, LEONI B, CAPONIO F, SANTAMARIA P. (2018). Nutritional characterization and shelf-life of packaged microgreens. *Food Funct,* **9**(11):5629-5640.

PATHAN S, SIDDIQUI RA. (2022). Nutritional composition and bioactive components in quinoa (chenopodium quinoa willd.) greens: a review. *Nutrients,* **14**(3):558.

PRITI, MISHRA GP, DIKSHIT HK, T V, TONTANG MT, STOBDAN T, SANGWAN S, ASKI M, SINGH A, KUMAR RR, TRIPATHI K, KUMAR S, NAIR RM, PRAVEEN S. (2021). Diversity in phytochemical composition, antioxidant capacities, and nutrient contents among mungbean and lentil microgreens when grown at plain-altitude region (delhi) and high-altitude region (leh-ladakh), ındia. *Front Plant Sci,* **12**:710812.

PUCCINELLI M, MALORGIO F, ROSELLINI I, PEZZAROSSA B. (2019). Production of selenium-biofortified microgreens from selenium-enriched seeds of basil. *J Sci Food Agric,* **99**(12):5601-5605.

RAO AP, PRADHAN AK, PATEL J. (2025). Persistence of foodborne bacterial pathogens on microgreens and soil irrigated with contaminated water. *J Food Prot,* 88(10):100594.

REED E, FERREIRA CM, BELL R, BROWN EW, ZHENG J. (2018). Plant-microbe and abiotic factors influencing salmonella survival and growth on alfalfa sprouts and swiss chard microgreens. *Appl Environ Microbiol,* **4**(9):e02814-17.

REBOLLEDO P, CARRASCO G, MOGGIA C, GAJARDO P, SANT'ANA GR, FUENTES-PENAILILLO F, URRESTARAZU M, VENDRUSCOLO EP. (2024). Assessment of vegetable species for microgreen production in unheated greenhouses: yield, nutritional composition, and sensory perception. *Plants (Basel)*, 13(19):2787.

RENNA M, CASTELLINO M, LEONI B, PARADISO VM, SANTAMARIA P. (2018). microgreens production with low potassium content for patients with impaired kidney function. *Nutrients,* **10**(6):675.

SALEH R, GUNUPURU LR, LADA R, NAMS V, THOMAS RH, ABBEY L. (2022). Growth and biochemical composition of microgreens grown in different formulated soilless media. *Plants (Basel),* 1**1**(24):3546.

SAMUOLIENĖ G, BRAZAITYTĖ A, VIRŠILĖ A, MILIAUSKIENĖ J, VAŠTAKAITĖ-KAIRIENĖ V, DUCHOVSKIS P. (2019). Nutrient levels in brassicaceae microgreens increase under tailored light-emitting diode spectra. *Front Plant Sci,* **10**:1475.

SHAHKOOMAHALLY S, ORTIZ I, ZHU X, TURNER ER, LI Y, SUN J, YANG T. (2025). Effect of low light ıntensity with supplemental far-red light on growth, yield and quality of broccoli microgreens. *Food Sci Nutr,* 13(7):e70542.

SHARMA S, SHREE B, SHARMA D, KUMAR S, KUMAR V, SHARMA R, SAINI R. (2022). Vegetable microgreens: the gleam of next generation super foods, their genetic enhancement, health benefits and processing approaches. *Food Res Int,* **155**:111038.

SILVA M, DOMINGUEZ-PERLES R, MORENO DA, VIEGAS O, FARIA MA, CARVALHO SMP, ISABEL IMPLVOF. (2025). Digestion of organosulfur compounds of two radish microgreen cultivars grown under different light treatments. *Food Res Int*, 217:116831.

SILVA MD, VASCONCELOS JM, DA SILVA FB, BAİLAO ASO, GUEDES IMR, VILELA MDS, COSTA AC, ROSA M, SILVA FG. (2024). Growing in red: impact of different light spectra and lighting conditions on lentil microgreens growth in vertical farming. *Front Plant Sci*, 15:1515457.

SINGH M, NARA U, RANI N, PATHAK D, KAUR K, SANGHA MK. (2022). Comparison of mineral composition in microgreens and mature leaves of celery (apium graveolens l.). *Biol Trace Elem Res,* **1**:1-11.

SKOWRON E, TROJAK M, PACAK I, WEZIGOWSKA P, SZYMKIEWICZ J. (2025). Enhancing the quality of indoor-grown basil microgreens with low-dose uv-b or uv-c light supplementation. *Int J Mol Sci*, 26(5):2352.

SOSNOWSKA D, ZAKLOS-SZYDA M, KAJSZCZAK D, PODSEDEK A. Bioactive properties and phenolic profile of bioaccessible and bioavailable fractions of red radish microgreens after in vitro digestion. *Molecules,* 30(14):2976.

SUN J, KOU L, GENG P, HUANG H, YANG T, LUO Y, CHEN P. (2015). Metabolomic assessment reveals an elevated level of glucosinolate content in CaCl₂ treated broccoli microgreens. *J Agric Food Chem,* **63**(6):1863-1868.

TALLEI TE, KAPANTOW NH, NIODE NJ, HESSEL SS, SAVITRI M, FATIMAWALI F, KANG S, PARK MN, RAIHAN M, HARDIYANTI W, NAINU F, KIM B. (2025). Integrative in silico and in vivo drosophila model studies reveal the anti-inflammatory, antioxidant, and anticancer properties of red radish microgreen extract. *Sci Rep,* 15(1):18533.

TENG J, LIAO P, WANG M. (2021). The role of emerging micro-scale vegetables in human diet and health benefits-an updated review based on microgreens. *Food Funct,* **12**(5):1914-1932.

TENG Z, LUO Y, PEARLSTEIN DJ, WHEELER RM, JOHNSON CM, WANG Q, FONSECA JM. (2023). Microgreens for home, commercial, and space farming: a comprehensive update of the most recent developments. *Annu Rev Food Sci Technol,* **14**:539-562.

THONGTIP A, MOSALEEYANON K, JANTA S, WANICHANANAN P, CHUTIMANUKUL P, THEPSILVISUT O, CHUTIMANUKUL P. (2024). Assessing light spectrum impact on growth and antioxidant properties of basil family microgreens. *Sci Rep,* 14(1):27875.

TİLAHUN S, BAEK MW, AN KS, CHOI HR, LEE JH, TAE SH, PARK DS, HONG JS, JEONG CS. (2024). Preharvest methyl jasmonate treatment affects the mineral profile, metabolites, and antioxidant capacity of radish microgreens produced without substrate. *Foods,* **4**;13(5):789.

WANG Q, KNIEL KE. (2015). Survival and transfer of murine norovirus within a hydroponic system during kale and mustard microgreen harvesting. *Appl Environ Microbiol,* **82**(2):705-13.

WEBER CF. (2017). Broccoli microgreens: a mineral-rich crop that can diversify food systems. *Front Nutr,* **4**:7.

WRIGHT KM, HOLDEN NJ. (2018). Quantification and colonisation dynamics of escherichia coli O157:H7 inoculation of microgreens species and plant growth substrates. *Int J Food Microbiol****,* 273**:1-10.

XIAO Z, BAUCHAN G, NICHOLS-RUSSELL L, LUO Y, WANG Q, NOU X. (2015). Proliferation of escherichia coli o157:h7 in soil-substitute and hydroponic microgreen production systems. *J Food Prot,* **78**(10):1785-90.

XIAO Z, NOU X, LUO Y, WANG Q. (2014). Comparison of the growth of escherichia coli o157: h7 and o104: h4 during sprouting and microgreen production from contaminated radish seeds. *Food Microbiol,* **44**:60-63.

XIAO Z, LESTER GE, LUO Y, WANG Q. (2012). Assessment of vitamin and carotenoid concentrations of emerging food products: edible microgreens. *J Agric Food Chem,* **60**(31):7644-51.

ZOU L, TAN WK, DU Y, LEE HW, LIANG X, LEI J, STRIEGEL L, WEBER N, RYCHLIK M, ONG CN. (2021). Nutritional metabolites in brassica rapa subsp. chinensis var. parachinensis (choy sum) at three different growth stages: microgreen, seedling and adult plant. *Food Chem,* **357**:
